# Child posttraumatic stress symptoms in an acute injury sample: Patterns of associations among child report, parent report, and child heart rate parameters

**DOI:** 10.1002/jts.22913

**Published:** 2023-02-14

**Authors:** Megan Bailey, Richard Meiser‐Stedman, Rachel Hiller, Katharina Haag, Sarah Lobo, Sarah L. Halligan

**Affiliations:** ^1^ Department of Psychology University of Bath Bath United Kingdom; ^2^ Department of Clinical Psychology and Psychological Therapies Norwich Medical School University of East Anglia United Kingdom; ^3^ Division of Psychology & Language Sciences University College London London United Kingdom; ^4^ Anna Freud National Centre for Children & Families London United Kingdom; ^5^ Department of Psychiatry and Mental Health University of Cape Town Cape Town South Africa

## Abstract

Parent–child agreement on measures of child posttraumatic stress disorder (PTSD) is moderate at best, and understanding of this discrepancy is limited. To address this, we conducted an item‐level investigation of parent–child symptom agreement to examine the potential influence of parental posttraumatic stress symptoms (PTSS) on parents’ reports of their child's PTSS. We also examined heart rate (HR) indices as possible independent indicators of child PTSD, examining patterns of association with parent versus child report. Parent–child dyads (*N* = 132, child age: 6–13 years, 91.7% White) were recruited after the child's hospital admission following an acute, single‐incident traumatic event. At 1‐month posttrauma, questionnaires assessing children's PTSS (self‐ and parental reports) and parental PTSS were administered. For a subset of participants (*n* = 70), children's HR recordings were obtained during a trauma narrative task and analyzed. Parent and child reports of child PTSS were weakly positively correlated, *r* = .25. Parental PTSS were found to be stronger positive predictors of parental reports of child PTSS than the children's own symptom reports, β = 0.60 vs. β = 0.14, and were associated with higher parent‐reported child PTSS relative to child reports. Finally, children's self‐reported PTSS were associated with HR indices, whereas parent reports were not, βs = −.33–.30 vs. βs = −.15–.01. Taken together, children's self‐reported PTSS could be a more accurate reflection of their posttrauma physiological distress than parent reports. The potential influence of parental PTSS on their perceptions of their child's symptoms warrants further consideration.

## 抽象

Traditional and Simplified Chinese Abstracts by the Asian Society for Traumatic Stress Studies

(AsianSTSS)

簡體及繁體中文撮要由亞洲創傷心理研究學會翻譯

Traditional Chinese

**急性損傷樣本中的兒童創傷後壓力症狀**:

信息提供者報告和兒童心率參數之間的關聯模式

摘要

對於兒童創傷後壓力症PTSD的測量方法, 父母與孩子的共識充其量是中等程度的, 對這種差異的理解是有限的。為了解決這個問題, 我們對親子症狀的一致性進行了項目層面的調查, 研究父母的創傷後壓力症 (PTSS) 對父母報告其孩子的PTSS的潛在影響。我們還研究了作為兒童創傷後壓力症可能的獨立指標的心率 (HR) 指數, 研究了與父母和兒童報告的關聯模式。親子關係 (N = 132, 兒童年齡:6‐13歲, 91.7%為白人) 是在兒童在急性、單一事件創傷後入院後被招募的。在創傷後1個月, 對兒童的PTSS (自我和父母的報告) 和父母的PTSS進行問卷調查。對於一部分參與者 (n = 70) , 在進行創傷敘述任務時獲得了兒童的心率記錄並進行了分析。父母和兒童對兒童PTSS的報告呈弱正相關, r = 0.25。發現父母的創傷後壓力症比兒童自己的症狀報告對父母的兒童創傷後壓力症有更強的正向預測作用, = 0.60 vs. = 0.14, 並且與父母報告的兒童PTSS相對於兒童報告的更高有關。最後, 兒童自我報告的PTSS與HR指數有關, 而父母的報告則沒有, s = ‐.33‐.30 vs. s = ‐.15‐.01。綜上所述, 兒童自我報告的創傷後壓力症可能比父母報告的更準確地反映了他們創傷後的生理痛苦。父母創傷後壓力症對他們對孩子症狀的看法的潛在影響值得進一步考慮。

**急性损伤样本中的儿童创伤后压力症状**:

信息提供者报告和儿童心率参数之间的关联模式

对于儿童创伤后压力症PTSD的测量方法, 父母与孩子的共识充其量是中等程度的, 对这种差异的理解是有限的。为了解决这个问题, 我们对亲子症状的一致性进行了项目层面的调查, 研究父母的创伤后压力症 (PTSS) 对父母报告其孩子的PTSS的潜在影响。我们还研究了作为儿童创伤后压力症可能的独立指标的心率 (HR) 指数, 研究了与父母和儿童报告的关联模式。亲子关系 (N = 132, 儿童年龄:6‐13岁, 91.7%为白人) 是在儿童在急性、单一事件创伤后入院后被招募的。在创伤后1个月, 对儿童的PTSS (自我和父母的报告) 和父母的PTSS进行问卷调查。对于一部分参与者 (n = 70) , 在进行创伤叙述任务时获得了儿童的心率记录并进行了分析。父母和儿童对儿童PTSS的报告呈弱正相关, r = 0.25。发现父母的创伤后压力症比儿童自己的症状报告对父母的儿童创伤后压力症有更强的正向预测作用, = 0.60 vs. = 0.14, 并且与父母报告的儿童PTSS相对于儿童报告的更高有关。最后, 儿童自我报告的PTSS与HR指数有关, 而父母的报告则没有, s = ‐.33‐.30 vs. s = ‐.15‐.01。综上所述, 儿童自我报告的创伤后压力症可能比父母报告的更准确地反映了他们创伤后的生理痛苦。父母创伤后压力症对他们对孩子症状的看法的潜在影响值得进一步考虑。

## Resumen

JOTS 22‐0098.R3 sp/abs

Spanish Abstracts by Asociación Chilena de Estrés Traumático (ACET)

Síntomas de estrés postraumático infantil en una muestra de lesiones agudas: Patrones de asociaciones entre informes de los niños, informes de los padres y parámetros de la frecuencia cardíaca en los niños

TEPT INFANTIL EN UNA MUESTRA DE LESIONES AGUDAS

El acuerdo entre padres e hijos en las medidas de trastorno por estrés postraumático (TEPT) infantil es moderado en el mejor de los casos, y se dispone de una comprensión limitada de esta discrepancia. Para abordar esto, llevamos a cabo una investigación a nivel de ítems sobre el acuerdo de síntomas entre padres e hijos para examinar la posible influencia de los síntomas de estrés postraumático de los padres en los informes de los padres sobre los síntomas de estrés postraumático de sus hijos. También examinamos los índices de frecuencia cardíaca (FC) como posibles indicadores independientes de TEPT infantil, examinando los patrones de asociación entre los informes de los padres y los hijos. Se reclutaron díadas padre‐hijo (*N* = 132, edad de los niños: 6‐13 años, 91.7% blancos) después de la admisión hospitalaria del niño después de un evento traumático agudo y único. Un mes después del trauma, se administraron cuestionarios para evaluar los síntomas de TEPT de los niños (informes propios y de los padres) y los síntomas de estrés postraumático de los padres. Para un subconjunto de participantes (*n* = 70), se obtuvieron registros de FC de los niños durante una tarea narrativa sobre el trauma y se analizaron. Los informes de los padres y los hijos sobre los síntomas de TEPT de los niños se correlacionaron débilmente de manera positiva, *r* = .25. Se encontró que los síntomas de estrés postraumático de los padres eran predictores positivos más fuertes de los informes de los padres sobre los síntomas de TEPT de los niños que los informes de los propios niños, β = 0.60 vs β = 0.14, y se asociaron con informes de los padres sobre síntomas de TEPT más altos en comparación con los informes de los niños. Finalmente, los síntomas de TEPT informados por los propios niños se asociaron con índices de FC, mientras que los informes de los padres no lo hicieron, βs = ‐.33–.30 vs βs = ‐.15–.01. En conjunto, los síntomas de TEPT informados por los propios niños podrían ser un reflejo más preciso de su angustia fisiológica después del trauma que los informes de los padres. La posible influencia del TEPT de los padres en su percepción de los síntomas de sus hijos requiere una mayor consideración.

Meta‐analytic evidence suggests that between 10% and 20% of children who have been exposed to a traumatic event develop posttraumatic stress disorder (PTSD; Alisic et al., 2014). Although approximately half of the children who initially develop PTSD are likely to show significant reductions in symptom severity in the months following trauma exposure, the remainder develop persistent posttraumatic stress symptoms (PTSS; Hiller, Meiser‐Stedman, et al., 2016). Beyond the first 6 months posttrauma, further natural decline in PTSS is relatively unlikely (Hiller, Meiser‐Stedman, et al., 2016), with persistent PTSD having long‐lasting impacts on the wider mental health and functional abilities of children (e.g., Lewis et al., 2019). Appropriate measurement of PTSS in this initial period after trauma exposure is, therefore, critical to ensure that appropriate interventions can be implemented.

Structured diagnostic interviews are typically recognized as the gold standard of evidence‐based assessments. However, despite the comprehensiveness of structured interviews to yield objective data and evaluate a range of diagnoses, evidence suggests that most care providers do not use structured interviews in clinical settings (Whiteside et al., 2016), with one of the most cited justifications emphasizing that structured interviews are too resource‐intensive for clinical use (Aboraya, 2009). Such findings highlight the need for robust mental health assessments that are practical to administer in real‐world clinical settings. Rating scales form an important part of evidence‐based practice in clinical settings and can provide a practical, reliable, and valid method of identifying, measuring, and tracking symptoms (Silverman & Ollendick, 2005).

A multi‐informant approach has become the recommended method of assessing child mental health (Kraemer et al., 2003). For rating scales, the multi‐informant approach typically includes children's self‐reports alongside reports from parents, caregivers, and/or teachers. However, meta‐analytic data show only low‐to‐moderate informant agreement on reports of the mental health of children (e.g., *r* = .20–.60; De Los Reyes et al., 2015). Notably, Lapouse and Monk (1958) described a general trend of higher agreement between child and parent reports on observable symptoms of mental health problems (e.g., bed wetting) relative to less observable symptoms (e.g., mood changes) and noted that low agreement between informants was primarily due to children reporting the presence of a symptom or behavior that their parent did not endorse, an observation that has been widely replicated (e.g., Rescorla et al., 2013).

Similar observations have been reported in research that has specifically addressed informant agreement relative to children's PTSS. For example, Meiser‐Stedman et al. (2007) found parent–child agreement to be poor for acute stress disorder and fair for PTSD in a sample of 10–16‐year‐olds involved in a single‐incident trauma. More limited research has investigated parent–child agreement for the intrusion, avoidance, and hyperarousal symptom clusters of PTSD, but the available evidence is inconsistent, with studies variously reporting high parent–child agreement for the intrusion cluster but poor agreement for avoidance and hyperarousal (Stover et al., 2010); moderate parent–child agreement for intrusion and avoidance symptoms but low agreement for hyperarousal (e.g., Humphreys et al., 2015); or modest associations across all symptom clusters, with the strongest correlations observed for hyperarousal (e.g., Meiser‐Stedman et al. 2007). Further examination of the types of PTSS upon which children and parents typically agree could help illuminate which symptom reports provide the best insight into children's posttrauma mental health across informants.

Researchers have also investigated potential explanations for the poor agreement between informants, with parental mental health explored as a possible influential factor. However, whereas parental depression and anxiety have been found to predict the extent of informant discrepancy in reports of child emotional and behavioral problems (e.g., Najman et al., 2001), researchers investigating the potential influence of parental PTSS have reported mixed results. Humphreys et al. (2015) found that parental PTSS did not moderate the association between parent and child reports of child PTSS. By contrast, Clawson et al. (2013) found high levels of parental PTSS to be associated with stronger parent–child concordance in reports of the child's PTSS among child cancer patients but not a control group. In a third study, the opposite pattern emerged, with higher maternal PTSS predicting lower concordance in a sample of Indian tsunami survivors (Exenberger et al., 2019). Further examination of this issue in relation to child PTSS is warranted.

Independent, biological indicators of posttraumatic distress can potentially provide additional insight into what is captured by parent versus child reports of child PTSS. Heart rate (HR) elevations have been observed in association with PTSD and are thought to reflect the occurrence of exceptionally strong adrenergic activation during a traumatic event that is later reactivated by trauma‐related cues (Pitman, 1989). Consistent with this theory, adult research has found an elevated HR, both at rest and during exposure to trauma cues, to be a valid predictor of PTSD, albeit with significant heterogeneity (e.g., Morris et al., 2016). HR variability (HRV; i.e., the variation in time intervals between heartbeats) has also been examined. Low HRV has generally been linked with impaired functioning of the autonomic nervous system, which negatively affects the body's ability to cope with stressors (e.g., Kim et al., 2018). HRV in the high‐frequency band power (HFPB; i.e., 0.15–0.4 Hz) is believed to reflect the functions of the parasympathetic nervous system, whereas low‐frequency band power (LFBP; i.e., 0.04–0.15 Hz) HRV may capture a combination of both sympathetic and parasympathetic functions. In a recent meta‐analysis, Nagpal et al. (2013) found that for both HFBP and LFBP, adults diagnosed with PTSD had reduced resting HRV compared with controls.

There is less empirical evidence of associations between HR/HRV and PTSD in children. Resting HR indices measured shortly following hospital admission for trauma have been found to predict PTSD in children, albeit with a small effect (meta‐analytic weighted *r* = .18; Alisic et al., 2011). Research investigating child HR reactivity to trauma cues has reported more mixed results. The findings from two studies demonstrated no evidence of altered HR reactivity in children diagnosed with PTSD, but samples for these studies were extremely small (Jones‐Alexander et al., 2005; Kirsch et al., 2015). In contrast, three larger studies identified positive associations between HR and child trauma exposure (Scheeringa et al., 2004), PTSS (Haag et al., 2019), and PTSD diagnosis (Gray et al., 2018). Thus, there is accumulating evidence that physiological reactivity to trauma cues is a biological correlate of children's PTSS.

In sum, the agreement between self‐ and parental reports of child PTSS is moderate, at best, and understanding of the factors that contribute to this discrepancy is limited. Physiological correlates of PTSS, such as HR reactivity to trauma cues, potentially provide an independent indication of the extent to which informant reports capture underlying distress but have not been used previously in this way. We addressed these gaps through a cross‐sectional analysis of parent and child reports of child PTSS in a single‐incident trauma sample. First, in addition to confirming previous findings of modest agreement between parent and child reports of child PTSS in the sample, we conducted exploratory analyses of the agreement between informants on each of the PTSD symptom clusters and the 17 individual PTSD symptoms outlined in the *Diagnostic and Statistical Manual of Mental Disorders* (4th ed.; *DSM‐IV*; American Psychiatric Association, 1994). Second, we explored parental PTSS as a predictor of parent reports of child PTSS. Finally, we compared child and parent reports with regard to their associations with child HR reactivity to trauma cues. We have previously reported associations between reactivity in HR and HRV and children's self‐reported PTSS in the same sample (Haag et al., 2019). Here, in addition to examining parent reports, we included analyses at the symptom cluster–level to further explore the patterns of association with these physiological indicators of distress.

## METHOD

### Participants

Parent–child dyads were recruited from four hospital emergency departments (EDs) in England. Children aged between 6 and 13 years who had been admitted to the ED following an acute trauma, defined as any event involving serious injury, potential threat to life, or threat to the child's personal integrity, were eligible for inclusion. Exclusion criteria were traumatic brain injury; safeguarding concerns, particularly suspicion that the child's injury was due to deliberate self‐harm or parental harm; or developmental difficulties that precluded mainstream schooling.

In total, 132 parent–child dyads participated, 110 (83%) of whom were offered HR assessments; HR equipment was not available for the first 22 families. HR data were subsequently collected for 76 families (57.6%); 19 families (17.3%) did not wish to participate in the HR assessments, situational constraints precluded these assessments for seven families (6.4%), and HR recordings for eight families (7.3%) were wholly unusable due to noise. No differences in sex, objective trauma severity, child‐reported PTSS, or parent‐reported child PTSS were found between children who underwent HR assessments and those who did not, *p*s = .616–.728; however, children with available HR data tended to be older than those without (*M* = 10.05 years vs. *M* = 9.38 years), *p* = .053.

### Procedure

Participants were recruited as part of a longitudinal study (see Haag et al., 2019; Hiller et al., 2018), with assessments conducted at 1‐month, 3‐months, and 6‐months posttrauma. The current research focused on cross‐sectional analyses at 1‐month posttrauma because this was the only time point at which HR and HRV were measured. Ethical approval was obtained from the University of Bath (14‐035) and Oxford A NHS (13/SC/0599) Research Ethics Committees.

Eligible dyads were identified by staff from four hospital EDs in England and, with parental permission, were contacted by the research team. Parents provided informed consent and children assented to take part. Assessments were completed 1‐month posttrauma in the family home. Parents and children were assessed separately by trained researchers. The 3‐min baseline HR recording was obtained as questionnaire and interview measures were completed. HR was then recorded first during the child narrative task and then during the joint narrative task. Dyads were debriefed at the end of the session, and the research team supported the families in seeking referrals to mental health services where appropriate.

### Measures

#### Descriptive information

Demographic characteristics and information related to the child's traumatic experience were obtained from parent interviews and ED notes. Standard hospital triage ratings, completed by ED nurses upon child admission, provided objective trauma severity ratings, ranging from 1 (*immediate care required*) to 4 (*less urgent*; Mackway‐Jones et al., 2013).

#### Child PTSS

The child self‐report and parent‐report versions of the UCLA PTSD Reaction Index (PTSD‐RI; Steinberg et al., 2004) were used to obtain child PTSS scores at 1‐month posttrauma. The 20‐item PTSD‐RI assesses the 17 core PTSD symptoms outlined in the *DSM‐IV*. Items are rated on a scale of 0 (*none of the time*) to 4 (*most of the time*); an additional “don't know” option is included on the parent version and was used infrequently across items (*Mdn* = 0, range: 0–7). This widely used measure has demonstrated good internal consistency (Cronbach's αs = .88–.91) and has been shown to differentiate successfully between trauma‐exposed and non–trauma‐exposed children (Steinberg et al., 2013). In the present sample, Cronbach's alpha was .89 for child self‐report and .88 for parent report.

#### Child PTSD diagnostic status

Child PTSD diagnosis status was determined using the Anxiety Disorder Interview Schedule PTSD Module (ADIS‐PTSD; Silverman et al., 1996), which was administered by trained researchers to parents and children. The ADIS‐PTSD is a well‐validated diagnostic tool based on *DSM‐IV* criteria (see Brown & Barlow, 2001). Diagnostic interrater agreement was established for 25% of interviews (*k* = 1.00), and approximately every sixth interview was discussed at a consensus meeting.

#### Parental PTSS

Parental PTSS related to their child's traumatic experience were assessed 1‐month posttrauma using the *DSM‐IV* version of the Posttraumatic Diagnostic Scale (PDS; Foa et al., 1997), which includes items related to 17 symptoms. Parents were asked to indicate how much each symptom had caused them distress during the past 30 days, rating answers on a 4‐point Likert scale ranging from 0 (*not at all*) to 3 (*almost always*). The PDS has demonstrated high test–retest reliability (*k* = .74), convergent validity with similar measures of PTSS, and excellent internal consistency (Cronbach's α = .92; Foa et al., 1997). In the current sample, Cronbach's alpha was .94.

#### Trauma narrative tasks

At 1‐month posttrauma, families participated in two trauma narrative tasks: one in which the child was asked to provide an independent account of their traumatic experience and one in which they described the event together with their parent. Both tasks were recorded and had no time limit (child narrative duration: *M* = 6.2 min, joint narrative duration: *M* = 11.6 min). Standard instructions were provided for each task. Children and parents were asked to describe the traumatic experience, starting from just before the event happened, and include anything they felt was important. The child narration task always took place first and was conducted alone with a researcher. The researcher did not interrupt the child but provided basic prompts if they were struggling (e.g., “And then what happened?”). For the joint narrative task, dyads were left alone to describe the event together. When they were finished, a member of the research team provided the parent with 13 prompt cards containing questions about the child's thoughts and feelings during and after the event and asked the parent to go through these cards with the child (see Supplementary Materials for full instructions and prompts).

#### HR Assessments

The Bio Radio 150 User Unit (Cleveland Medical Devices Inc.; Cleveland, OH, USA) and BioCapture software (Biopac‐Systems Goleta, CA, USA) were used to collect electrocardiographic (ECG) data at a rate of 600 Hz during a 3‐min baseline procedure (i.e., as the child filled in ID numbers on study questionnaires) and during each of the two trauma narrative tasks. A disposable electrode was placed below each collarbone, approximately 6 in apart, and a ground electrode was placed on the elbow of the nondominant hand to limit noise due to movement, with the child also being instructed to sit as still as possible. ECG data were preprocessed using the ANSLAB (Version 2.6) software (Blechert et al., 2016). Recordings were then inspected for artifacts and ectopic heartbeats, and R‐peak markers set by the software were checked and corrected if necessary. Additionally, requirements of more than 120 s of baseline recording and more than 300 s of narrative recordings were established due to the sensitivity of HRV analyses to noise distortions (e.g., Bilchick & Berger, 2006). This resulted in the exclusion of three participants at baseline, one participant for the child narrative condition, and four participants for the joint narrative condition. Furthermore, sections of recording with more than 3 s of consecutive noise present were cut, and the longest analyzable segment was retained. Nine participants were affected by this at baseline, with approximately 78% of the recording retained; 10 were affected in the child narrative condition (77% retained), and 20 were affected in the joint narrative condition (74% retained). The mean HR for each condition was calculated after preprocessing, and nonparametric Fourier transformation was applied to extract the two HRV indices (i.e., HFBP and LFBP). Six segments were excluded in this final stage due to abnormal HR (*n* = 1) or HRV indices that were more than 3 standard deviations from the mean (*n* = 5), resulting in a final sample of 70 dyads with useable HR data (i.e., reactivity data comprising baseline and at least one narrative task).

### Data analysis

Data were analyzed using SPSS (Version 26.0) for Macintosh. PTSD‐RI and PDS scores were square root–transformed to address skewness. Examinations of HR, HFBP, and LFBP at baseline relative to during both the joint and child narrative tasks indicated that HR/HRV tended to be higher during the tasks compared to baseline, *p*s = <.001–.028, except mean HR during the joint narrative task, *t*(58) = 1.03, *p* = .308. Baseline and task HR parameters were all positively correlated, *rs* = .50–.90. Therefore, residualized change scores from baseline HR/HRV to narrative HR/HRV were calculated to control for the child's baseline HR. The resultant scores were normally distributed. For each HR index, the residualized change scores from the two narratives were averaged, as scores were highly correlated, *r*s = .58–.71, and were not significantly different across tasks, *p*s = .614–.856.

Intraclass correlations (i.e., two‐way mixed effects for single raters/absolute agreement) were used to assess the association between parent and child report of child PTSS scores (i.e., total scores and scores for the intrusion, avoidance, and hyperarousal clusters). Binary logistic regression was used to test the association between each informant's scores and child diagnostic status (PTSD diagnostic criteria met or not met). Agreement between parent and child reports on the 17 *DSM‐IV* PTSD symptoms was explored using Cohen's weighted kappa analyses using linear weightings for ordinal scores. Linear regression analysis was conducted to assess whether parents’ PDS scores predicted parent report of child PTSS. This analysis was repeated in two sensitivity analyses, adjusting for child self‐reported PTSS and child PTSD diagnostic status as potentially more objective indicators of distress. We also calculated the discrepancy between child and parent reports of child PTSS by subtracting the total parent report score from the total child report score, then used linear regression to test whether parental PDS scores predicted this discrepancy score. Finally, we used linear regression to investigate whether child or parent reports of child PTSS more strongly predicted child HR indices, with these analyses repeated for each PTSD symptom cluster. Child age, sex, and triage category were considered as potential control variables in all regression analyses, and bootstrapping was carried out with 10,000 replications to derive confidence intervals (CIs) around parameter estimates. The Benjamini–Hochberg procedure (Benjamini & Hochberg, 1995), with a false discovery rate set to 5%, was used to correct for multiple comparisons. All findings remained significant.

Data were missing for parent‐reported UCLA‐RI scores (*n* = 4), PDS scores (*n* = 9), and HR data (*n* = 62, as previously detailed). To address the resultant variability in sample sizes across our analyses, we conducted multiple imputation for most analyses. All findings remained the same when imputed versus complete data were used. We were unable to conduct multiple imputation for the HR data analyses due to the large amount of missingness and lack of additional information (e.g., longitudinal HR data) to inform the imputation model.

## RESULTS

### Descriptive statistics

The total sample comprised 132 children aged 6–13 years and their participating parent (90.2 % mothers, age range: 25–60 years). There were available HR data for 70 dyads. The primary reason for ED admission was experiencing a motor vehicle accident (55.9%). Detailed sample characteristics are provided in Table 1, and correlation coefficients between variables are provided in Table 2. We considered a priori child age, sex, and triage category, as an indicator of trauma severity, as possible covariates for the analyses. Sex was not related to any outcome variable and was not considered further. Age was inversely related to child self‐reported PTSS and child HR and was adjusted for in all regression models. In addition, triage category was positively associated with parental PTSS and was adjusted for in all models involving PDS scores.

**TABLE 1:** Descriptive information

**Variable**	***M***	***SD***	***n***	**%**
Parent characteristics				
Age (years)	39.70	7.0		
Child's mother			119	90.2
Married or cohabiting			97	73.5
Educational attainment				
School until ≤ 16 years of age			36	27.3
Further education (e.g., vocational training)			50	37.9
Higher education			46	34.8
Child characteristics				
Age (years)	9.77	1.99		
Male sex			82	62.1
Race/ethnicity				
White			121	91.7
Mixed			4	3.0
Asian			4	3.0
Caribbean or Black			1	0.8
Other			2	1.5
Trauma characteristics				
1 (immediate care required)			61	46.2
2 (very urgent)			29	22.0
3 (urgent)			26	19.7
4 (less urgent)			16	12.1
Mechanism of trauma				
Road traffic accident			75	55.9
Fall			25	18.9
Acute illness			10	7.6
Sporting injury			6	4.5
Assault			3	2.3
Other			11	8.3
Days in hospital	2.64	4.83		
Days of school missed	5.52	5.98		
Required ambulance or helicopter transport			90	68.2
Met PTSD diagnostic criteria at T1^a^			34	25.8
Child self‐reported PTSS at T1^b^	3.97	1.68		
Parent‐reported child PTSS at T1^b^	3.20	1.70		
Parent PTSS^c^	2.90	1.88		

*Note*: *N* = 132. PTSD = posttraumatic stress disorder; T1 = Time 1 (1‐month posttrauma); PTSS = posttraumatic stress symptoms.

^a^ Determined using the Anxiety Disorder Interview Schedule PTSD Module, which was administered to parents and children.

^b^ Assessed using the UCLA PTSD Reaction Index.

^c^ Assessed using the Posttraumatic Diagnostic Scale.

**TABLE 2:** Correlations between child age, child sex, child triage category, child posttraumatic stress symptom (PTSS) scores, parental PTSS scores, and heart rate (HR) indices

	1.	2.	3.	4.	5.	6.	7.	8.	9.	*n*
1. Child age	–	−.06	.06	−.20*	−.08	−.05	−.27*	.05	.20	132
2. Child sex		–	.00	.09	−.12	−.05	.11	.06	.12	132
3. Child triage category			–	.00	−.09	−.26**	.01	−.08	.20	132
4. Child‐reported PTSS				–	.29**	.27**	.34**	−.32**	−.37**	132
5. Parent‐reported PTSS					–	.62**	.16	−.24	−.19	128
6. Parental PTSS						–	.06	−.23	−.29*	123
7. Mean HR							–	−.53**	−.38**	70
8. HFBP								–	.72**	70
9. LFBP									–	70

*Note*: HFBP = high‐frequency band power; LFBP = low‐frequency band power.

*p < .05. **p < .01.

### Associations among child and parent reports of child PTSS and child PTSD diagnostic status

Parents reported lower child total PTSS scores than children (parent: *M* = 3.20, *SD* = 1.70; child: *M* = 3.95, *SD* = 1.67), *t*(127) = 4.19, *p* < .001, and lower scores on the intrusions (parent: *M =* 1.47, *SD =* 1.17; child: *M =* 1.82, *SD =* 1.35); *t*(131) = 2.54, *p* = .012; avoidance (parent: *M =* 1.56, *SD =* 1.16; child: *M =* 2.29, *SD =* 1.17), *t*(131) = 5.59, *p <*.001; and hyperarousal symptom clusters (parent: *M =* 1.94, *SD =* 1.10; child: *M =* 2.38, *SD =* 1.07), *t*(131) = 3.75, *p <*.001.

As expected, correlational analyses showed a small positive association between child and parent reports of child PTSS, intraclass *r =* .25, *p =* .001, 95% CI [.08, .40]. Associations were similarly small for the intrusion, intraclass *r =* .24, *p =* .002, 95% CI [.07, .39]; avoidance, intraclass *r =* .14, *p =* .025, 95% CI [−.01, .30]; and hyperarousal clusters, intraclass *r =* .25, *p =* .001, 95% CI [.08, .40]. Binary logistic regression analyses indicated that child‐ and parent‐reported symptoms were each independently associated with the likelihood of a child meeting the criteria for a PTSD diagnosis, but these associations were larger in magnitude for child, *B* = 1.11, versus parent reports, *B* = 0.72 (see Supplementary Table S1).

**FIGURE 1:**
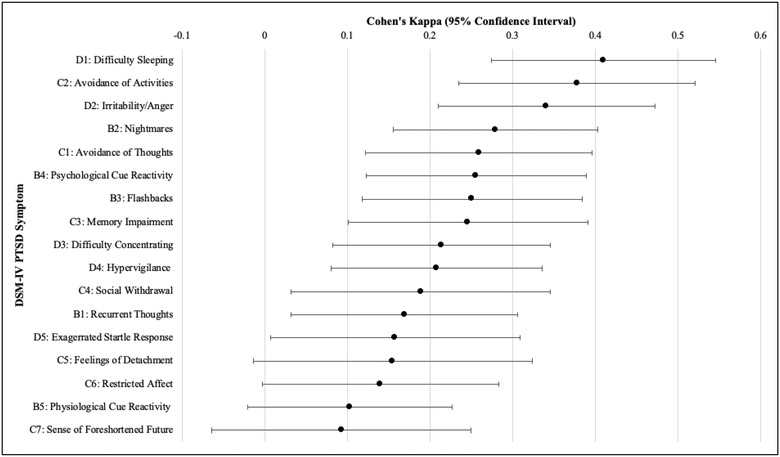
Forest plot of Cohen's kappa coefficients and 95% confidence intervals for parent–child posttraumatic stress disorder (PTSD) symptom report agreement *Note*: Symptoms reflect PTSD criteria outlined in the *Diagnostic and Statistical Manual of Mental Disorders* (4th ed.).

Exploratory Cohen's weighted kappa analyses to investigate parent–child agreement on each of the 17 *DSM‐IV* PTSD symptoms revealed poor‐to‐moderate agreement for all symptoms, κs = .09–.41 (Figure 1). Significant parent–child agreement was found for 15 of the 17 symptoms, although agreement was still relatively poor. Agreement between parents and children on items related to physiological cue reactivity (Symptom B5) and sense of foreshortened future (Symptom C7) was nonsignificant.

### Parental PDS scores as a predictor of parent‐reported child PTSS

Linear regression was used to test whether parental PTSS were predictive of their reports of their child's PTSS. Child age and triage category were included as control variables. Model 1 (see Table 3) predicted parent reports of child PTSS by parent PDS scores, child age, and child triage. In total, 40% of the variance in parent‐reported child PTSS was explained by predictors in the model, with parent PDS scores being the only positive predictor, *B* = 0.60, 95% CI [0.45, 0.74], β = .64. Two sensitivity analyses were run to examine whether this association was robust to adjustment for other indicators of child distress. First, a model that additionally included children's self‐reported PTSS accounted for 41% of the variance in parent reports of child PTSS, with parent PDS scores again the only contributor, *B* = 0.56, 95% CI [0.41, 0.71], β = .60. Second, a model adjusting for children's PTSD diagnosis based on clinical interview accounted for 47% of the variance in parent reports of child PTSS. Both parent PDS scores, *B* = 0.50, 95% CI [0.35, 0.64], and child diagnostic status, *B* = 1.17, 95% CI [0.50, 1.89], contributed to the model, although parent PDS scores, β = 0.53, were more strongly associated with parent‐reported child PTSS than child diagnostic status, β = 0.30. See Supplementary Table S2 for the full results of both models.

**TABLE 3:** Regression analyses for parent posttraumatic stress disorder (PTSD) symptoms as a predictor of parent‐reported child symptom scores (Model 1) and discrepancy scores between parent and child reports of child symptoms (Model 2)

**Predictor**	***B*** **^a^**	**95% CI**	***SE***	**β**	***p***
Model 1: Parent‐reported child PTSS					
Parent PDS score	0.60	[0.45, 0.74]	0.07	.64	< .001
Child age	−0.04	[−0.18, 0.08]	0.07	−.05	.515
Child triage category	0.15	[−0.07, 0.38]	0.12	.10	.200
	*F*(3, 115) = 25.08, *p* < .001, *R* ^2^ = .40				
Model 2: Child‐parent discrepancy scores^b^					
Parent PDS score	−0.33	[−0.52, −0.13]	0.10	−.31	.001
Child age	−0.11	[−0.29, 0.07]	0.09	−.11	.226
Child triage category	−0.05	[−0.38, 0.29]	0.18	−.03	.783
	*F*(3, 115) = 4.10, *p* = .008, *R* ^2^ = .10				

*Note. n* = 118. PDS = Posttraumatic Stress Diagnostic Scale; PTSS = posttraumatic stress symptoms; CI = confidence interval.

^a^ Bootstrapped with 10,000 replications.

^b^ Discrepancy scores computed as child self‐reported symptoms minus parent‐reported child symptoms.

Finally, a linear regression analysis was conducted to examine whether parental PTSS were predictive of the discrepancy between child‐ and parent‐reported child PTSS scores, coded as child scores minus parent scores, after adjusting for child age and triage category. The resultant model explained 10% of the variance in discrepancy scores (see Table 3, Model 2). Parent PDS scores were found to be negatively associated with discrepancy scores, *B* = −0.33, 95% CI [−0.52, −0.13], *β* = −.30, indicating that higher levels of parental symptoms were associated with a higher tendency for parental ratings of children's symptoms to exceed children's own ratings. Following reviewer feedback, we also ran this analysis using symptom‐level agreement scores, calculated as the total number of symptoms for which parents and children gave the same rating. Conclusions were the same, with higher parental PTSD associated with lower parent–child agreement scores, *B* = −0.043, 95% CI [−0.064, −0.021], *β* = −.34, in a regression adjusted for child age and triage category.

### Associations between child reports, parent reports, and child HR indices

Three linear regression analyses were conducted to investigate whether child or parent reports of total child PTSS showed stronger associations with child HR indices, averaged across two narrative trauma recall tasks, adjusted for child age. As presented in Table 4, child self‐reported PTSS contributed to the prediction of HR (Model 1), HFBP (Model 2), and LFBP (Model 3), whereas parent reports of child PTSS failed to contribute to the models.

**TABLE 4:** Regression analyses of child and parent reports of child posttraumatic stress symptoms (PTSS) as predictors of child heart rate (HR) indices

**Predictor**	***B***	**95% CI** **^a^**	***SE***	**β**	***p***
Model 1: Mean HR					
Child‐report PTSS	0.15	[0.02, 0.28]	0.06	.30	.020
Parent‐report PTSS	0.01	[−0.01, 0.14]	0.06	.01	.915
Child age	−0.09	[−0.20, 0.02]	0.05	−.19	.119
	*F*(3, 64) = 4.26, *p* = .008, *R* ^2^ = .17				
Model 2: HFBP					
Child‐report PTSS	−0.16	[−0.29, −0.03]	0.07	−.31	.020
Parent‐report PTSS	−0.07	[−0.20, 0.03]	0.06	−.15	.244
Child age	−0.03	[−0.14, 0.09]	0.06	−.07	.600
	*F*(3, 64) = 3.25, *p* = .027, *R* ^2^ = .13				
Model 3: LFBP					
Child‐report PTSS	−0.18	[−0.30, −0.07]	0.07	−.33	.011
Parent‐report PTSS	−0.03	[−0.15, 0.10]	0.06	−.06	.648
Child age	0.04	[−0.08, 0.15]	0.06	.10	.473
	*F*(3, 64) = 3.89, *p* = .013, *R* ^2^ = .15				

*Note*. *n* = 68. HFBP = high‐frequency band power; LFBP = low‐frequency band power; CI = confidence interval.

^a^ Bootstrapped with 10,000 replications.

In exploratory analyses, we reran the previously described regression models separately for each of the PTSD symptom clusters. Full‐model results can be found in Supplementary Tables S3–S5. Child‐reported, but not parent‐reported, hyperarousal symptoms were found to be predictive of child HR, *B =* 0.25, 95% CI [0.05, 0.44], β = .30; HFPB, *B =* −0.14, 95% CI [−0.51, −0.14], β = −.36; and LFPB, *B =* −0.24, 95% CI [−0.43, −0.07], β = −.28. In addition, child‐reported, but not parent‐reported, intrusion symptoms were associated with LFPB reactivity, *B =* −0.23, 95% CI [−0.39, −0.08], β = −.35, but not other HR parameters. Child‐ and parent‐reported avoidance symptoms were not associated with any HR outcome.

## DISCUSSION

Consistent with previous research, we found poor‐to‐moderate parent–child agreement on child PTSS, with children reporting significantly more PTSS than parents across the intrusion, avoidance, and hyperarousal symptom clusters. Parental PTSS positively predicted reports of child PTSS, even when controlling for child self‐reported symptoms or the child's PTSD diagnostic status, and were also predictive of a higher discrepancy between child and parent reports of children's symptoms. Child self‐reported PTSS were associated with HR indices, particularly symptoms of hyperarousal, whereas parent reports of child PTSS did not account for variance in the same indices.

Parents were found to underreport child PTSS relative to their children's self‐reported symptoms, and parent and child reports of child PTSS were only weakly positively correlated in this study, aligning with the results of previous research (e.g., Meiser‐Stedman et al., 2017). Additionally, we found that child self‐reported symptoms were more strongly associated with PTSD diagnostic status than parent reports. Regarding assessment items related to individual symptoms, exploratory analyses found poor‐to‐moderate agreement for all items, with no clear pattern emerging. Notably, the intrusion, avoidance, and hyperarousal symptom clusters appeared to have similar ranges of agreement. Additionally, there did not appear to be any distinction between external (i.e., observable) and internal (i.e., nonobservable) symptoms. This preliminary finding is inconsistent with prior research addressing parent–child agreement on general child mental health, in which researchers have observed that parent–child agreement tends to be higher for observable symptoms compared to nonobservable symptoms (e.g., De Los Reyes et al., 2015). It is possible that PTSS overall do not manifest sufficiently clearly in children for parents to be able to detect them reliably, at least in samples characterized by relatively mild symptoms, like the current sample.

In terms of contributors to discrepancies in parent–child agreement, we found that parental PTSS were potentially important. Parents’ PTSS predicted parent reports of child PTSS even after controlling for children's self‐reported symptoms or child PTSD diagnostic status. These findings suggest that parents with higher levels of PTSS may report more symptoms in their children compared to parents with lower PTSS regardless of their child's self‐reported symptom levels or actual PTSD diagnostic status. In addition, the extent of the discrepancy between parent and child reports was associated with parental PTSD symptoms such that higher levels of parental distress predicted high parental ratings of child symptoms relative to child self‐ratings. Overall, these findings suggest that parents’ perceptions of their children's PTSS may, at least in part, be shaped by their own trauma‐related symptoms. This is broadly consistent with previous observations that parental emotional impairment is positively associated with parental reports of child emotional and behavioral problems relative to the self‐reports of children (e.g., Najman et al., 2001). It is worth noting that despite these findings, the association between parental PTSS and parent‐reported ratings of child PTSS also likely reflects other factors. For instance, there is a well‐established association between child and parental PTSS (Morris et al., 2016), which could be a consequence of reciprocal influences between parent and child distress and underlying factors, such as a shared genetic vulnerability (Bomyea et al., 2012) or trauma severity (Hiller, Halligan, et al., 2016). With respect to the latter, it is notable that in the present sample, more severe child trauma at ED admission, based on triage category, was associated with higher levels of parental PTSS.

We examined the patterns of association between child and parent reports of child PTSS with child HR/HRV, as averaged across two narrative trauma recall tasks, which are physiological markers that have previously demonstrated correlations with PTSD. We previously reported in the same sample that HR reactivity during a trauma exposure task was positively correlated, and HFBP and LFBP were both negatively correlated, with child‐reported PTSS (Haag et al., 2019). In contrast, we found no evidence that parental reports of child PTSS were associated with any HR marker in the current study. When child and parent reports of overall child PTSS were examined simultaneously in regression models, only child‐reported scores were independently associated with all three HR indices. We also examined the *DSM‐IV* intrusion, avoidance, and hyperarousal symptom clusters and found that HR indices were consistently associated with children's reports of hyperarousal symptoms. Overall, these observations suggest that children, but not parents, may be attuned to elements of children's posttraumatic distress that are reflected in their autonomic reactivity to trauma cues. Consequently, relying only on parental reports of children's PTSS could fail to detect potentially significant aspects of children's symptom profiles. Nonetheless, the current findings require replication given that links between HR reactivity and PTSD are still being established in the children's trauma field and the availability of HR data in the current sample was limited.

The current findings highlight numerous factors for consideration regarding the evidence‐based assessment of child PTSD, particularly pertaining to the balancing of strong psychometric properties with practicality when using these assessments in clinical settings. Diagnostic interviews may not always be suitable in clinical or research contexts. For instance, diagnostic interviews are often unsuitable for contexts that require large‐scale screening (e.g., postdisaster), and when the prevalence of PTSD is expected to be low. In these situations, tools designed to measure PTSS rather than evaluate diagnostic status are likely to provide a more sensitive assessment. Additionally, care providers have indicated that structured interviews can be too resource‐intensive or require additional training that is not always accessible (Aboraya, 2009; Whiteside et al., 2016). Given this, an understanding of what can be learned from self‐ and parent‐report measures of PTSS is important. The current observations are consistent with the view that children's self‐reported PTSS are important in providing an accurate picture of child distress and that parent report alone could result in the underdetection of child PTSD. Research by Scheeringa et al. (2006) supports this suggestion, reporting an 8.9‐fold increase in PTSD diagnosis when both child and parent report of child PTSS were considered (37.5%) compared to only parent reports (4.2%). For the benefit of clinical practice, future research should investigate further the contributors to poor informant agreement regarding child PTSS. In the present study, we found that parents’ own PTSS were associated with their reports of their children's PTSS, and this is one area that could be usefully explored. For example, parents may find it helpful to understand that their child might not share their own distress related to the child's traumatic experience and could be supported in gaining more objective information about how their child is coping. The latter may be a particularly important consideration for assessing the mental health of children who may be unable or unwilling to provide symptom self‐reports.

The present study has several strengths, including the use of both parent and child informants and assessing both HR and HRV indices. Nevertheless, there are also some limitations. First, over 90% of the sample was White, which limits the ability to generalize the findings to populations that were not adequately represented in this research. Moreover, all children in the current sample were exposed to a single‐incident trauma, mainly accidental injuries. Levels of PTSS in the present sample were also relatively low overall, with only 25.8% of the sample (*n* = 34) meeting the criteria for PTSD, and, as a result, the findings may not generalize to other trauma populations (e.g., child cancer patients; see Clawson et al., 2013). Second, we did not control for prior trauma exposure and preexisting PTSD. Although our analyses focused on symptom presentations after a specific traumatic event, it is possible that informant reports were influenced by prior trauma or trauma‐related distress. Third, only about half of the participants in the study completed the HR analyses, and the subsample of children who underwent HR assessments tended to be slightly older than other participants. Additionally, the current findings averaged HR/HRV across two trauma narrative tasks relative to resting baseline measurements, and these scores may not specifically reflect reactivity to trauma cues. Replicating the current HR findings in a larger sample and including comparisons to a nontrauma narrative task is essential. Finally, the inclusion of further potentially relevant covariates (e.g., amount of talking during the narrative tasks) could also strengthen analyses of physiological reactivity.

In sum, the present study expanded upon previous research investigating the multi‐informant approach to assessing child PTSS following trauma exposure. We evidenced low‐to‐moderate parent–child agreement on overall and clustered PTSS. Additionally, the findings evidence the potential role that parental distress can have on their reports of children's PTSS. Finally, to our knowledge, this is the first contribution to the literature to include an evaluation of the patterns of association between child‐reported PTSS, parent‐reported child PTSS, and child HR indices that demonstrates stronger associations between child report and child HR indices compared to parent report. We note the clinical relevancy of these findings, particularly in relation to the utility of self‐report in clinical practice. Additionally, the study highlights that although parental distress is certainly not the only factor that may influence parent reports of child symptoms, it is one that can potentially be addressed. For instance, clinicians and researchers can provide parents with relevant information and guidance to help them understand that their child may not share the distress they themselves are feeling about their child's traumatic experience. Finally, this study establishes a strong base upon which parents can seek guidance about how best to support their children following exposure to a potentially traumatic event.

## OPEN PRACTICES STATEMENT

The study reported in this article was not formally preregistered. For data access, see Halligan and Hiller (2017), “The Role of Trauma‐Specific Behaviours and Parenting Style in Facilitating Child Psychological Adjustment” [Data Collection]. Colchester, Essex: UK Data Archive https://doi.org/10.5255/UKDA‐SN‐852668

## AUTHOR NOTE

This research was funded by an Economic and Social Research Council grant (ES/ K006290/1) awarded to Sarah L. Halligan. The authors have no relevant financial or non‐financial interests to disclose.

The authors wish to thank the children and parents who participated in this study and the emergency department staff at Bristol Royal Hospital for Children, Royal United Hospital Bath, Great Western Hospital, and Gloucestershire Hospital.

## Supporting information

Supplementary Materials

Table Information
