# Supplementary material for: Child posttraumatic stress symptoms in an acute injury sample: Patterns of associations among child report, parent report, and child heart rate parameters
Source: J Trauma Stress. 2023 Feb 14;36(2):333–45. doi: 10.1002/jts.22913 (PMC10946953; doi:10.1002/jts.22913)
Supplement: Supplementary file 1 — Supplementary Materials [file JTS-36-333-s002.docx]

**CHILD TRAUMA NARRATIVE PROCEDURE**

General Procedure:

1. Child rates *current level* of distress and agitation
2. Narrative (child tells as much as they would like, and assessor only uses basic prompts)
3. Once the child has given narrative refer to ‘after narrative’ prompts to gather information on thoughts and feelings before, during, and after accident
4. Child uses rating scales to rate distress and agitation felt whilst they recalled the event to the assessor, recalls most upsetting part of event, rates distress and agitation at worst point of event.

**Prior to narrative:**

Child rates current emotional state

(not distressed 🡨🡪 very distressed [scale 1] AND relaxed🡨🡪agitated/stressed [scale 2]), on 0-10 point visual analogue scales

*Before we start I’d like to get an idea of how you’re feeling now. We’re going to use these scales* [face scales] *to help you rate how you are feeling. The first feeling I’m going to ask you about is how distressed/upset you feel* [show scale 1]*. On this scale you can rate your feeling from* [point to ‘0’] *not distressed/upset at all to* [point to ‘5’] *somewhat distressed/upset to* [point to ’10’] *extremely distressed/upset.*

*If I asked you how distressed/upset you are feeling right now from not distressed/upset at all* [point to ‘0’] to *extremely distressed/upset* [point to ‘10’] *where would you be?*

[Present scale 2]*What about if we used this scale to rate how relaxed/stressed you are feeling from* [point to ‘0’] *relaxed to* [point to ‘5’] *somewhat stressed/agitated to* [point to ‘10’] *extremely stressed/agitated, like your body feels tense.*

*How would you rate how you’re feeling right now?*

**Narrative Instructions for the child:**

“I understand that you experienced ………………………………………………………..I would like you to think back to that time and tell me everything you can about what happened. If you can, include what you saw, heard, thought and how you felt at the time. Begin just before the…………………………..and talk about as much of that day as you like, including what happened at the hospital and afterwards if you want to. I’m going to sit quietly and let you talk for as long as you like.”

Example prompts for *during* the child’s narrative (if required)

- And then what happened?
- Can you tell me more about that?
- I’ll let you think for a few moments in case there is anything more you want to tell me.
- Go on if you can.

AFTER the child cannot provide any further information, based on the above prompts, go through the following list of prompts:

- *I would like you to think back to just before the accident. Can you tell me how you were feeling just before the event?*
- *Thinking about during the event* (if they can remember). *Can you tell me a bit about how you were feeling? Were there any thoughts going through your head? (Ask as discrete questions.)*
- *Thinking about after the event, like when you were in hospital – Can you tell me how you were feeling? Were there any thoughts going through your head? (Ask as discrete questions.)*

**Rating scales for after the recall:**

1. “Now we’re going to use the same ratings scales we used earlier. I want to know how you felt re-telling me the story of your accident.”
   - [Present with scale 1 - distress]*How would you rate how distressed/upset you felt when you were telling me about the event? From not at all distressed to extremely upset.*
   - [Present with scale 2 - stressed] *How would you rate how relaxed or stressed you felt when you were telling me about the event? From relaxed to extremely agitated/stressed, like feeling tense in your body?*
2. “Which part do you think was the most upsetting or scary for you?”
3. Child rates emotional state at the worst point of the event.
   - (not distressed 🡨🡪 very distressed [scale 1] AND relaxed🡨🡪agitated/stressed [scale 2]), on 0-10 point visual analogue scales

**JOINT TRAUMA NARRATIVE PROCEDURE**

**Prior to start:**

Parent and child rate their current emotional state

(not distressed 🡨🡪 very distressed [scale 1] AND relaxed🡨🡪agitated/stressed [scale 2]) on 0-10 point visual analogue scales. (*Parent to write down.)*

**Instructions for parent and child:**

“We would now like you to talk to each other about the frightening event/ accident that happened and try to describe it together. You can do this in any way that you want; there are no rules. Try to begin just before the event happened, and include anything about what happened afterwards that you think is important. We will leave you to talk for as long as you like. When you have finished describing the event as much as you want in your own words please call us back and we will give you some cards with some extra questions to look at.”

LEAVE ROOM AND WAIT UNTIL CALLED BACK IN BEFORE GIVING PROMPT CARDS

ENSURE PARTICIPANTS HAVE BEEN TALKING FOR AT LEAST 1 MINUTE BEFORE GIVING CARDS – IF CALLED SOONER, GIVE A BASIC PROMPT SUCH AS “WE’LL JUST GIVE YOU A LITTLE LONGER TO SEE IF THERE IS ANYTHING YOU CAN THINK OF TO ADD. WE’LL BRING THE CARDS INTO THE ROOM IN A SHORT WHILE.”

**Prompt cards:**

1. What was happening just before the event?
2. What happened after the accident and before you arrived at hospital?
3. How did you feel at the time (of the event)?
4. Did you also notice any feelings in your body?
5. What sorts of things were going through your mind during the frightening event?
6. Who else was there and what were they doing?
7. How well were you able to cope at the time (of the event)?
8. What was happening when you were at the hospital?
9. How did you feel when you were in the hospital?
10. What sorts of things were going through your mind while you were at the hospital?
11. Who else was there and what were they doing, while you were at the hospital?
12. Do you feel like you’ve changed since what happened?
13. Are there any things you wouldn’t do anymore since what happened?

**After the recall:**

Parent and child each rate their emotional state while describing what happened

on 0-10 point visual analogue scales emotional state (not distressed 🡨🡪 very distressed [scale 1] AND relaxed🡨🡪agitated/stressed [scale 2]) (*Parent to write down*.)
