# Supplementary material for: Child posttraumatic stress symptoms in an acute injury sample: Patterns of associations among child report, parent report, and child heart rate parameters
Source: J Trauma Stress. 2023 Feb 14;36(2):333–45. doi: 10.1002/jts.22913 (PMC10946953; doi:10.1002/jts.22913)
Supplement: Supplementary file 2 — Table Information [file JTS-36-333-s001.docx]

**SUPPLEMENTARY MATERIALS**

**Table 1.** *Binary logistic regression (n=128): prediction of child PTSD diagnostic status (criteria for diagnosis met versus not met) from child reported and parent reported child PTSS*

| **Predictor** | ***B* (95% CI)^a^** | ***SE*** | **Wald** | ***p*** | **Exp(B) (95% CI)** |
| --- | --- | --- | --- | --- | --- |
| Child PTSD Diagnostic Status; $\chi$*^2^*(2)=64.34, *p*=<.001, Nagelkerke *R^2^*=.59 | | | | | |
| CR Child PTSS | 1.11 (0.75, 1.81) | 0.25 | 19.06 | <.001 | 3.03 (1.84, 4.98) |
| PR Child PTSS | 0.72 (0.39, 1.28) | 0.19 | 14.33 | <.001 | 2.05 (1.41, 2.96) |

^a^ CI are bootstrapped with 10,000 replications

*Note*. CR: Child report. PR: Parent report. SE: Standard error.

**Table 2.** *Regression analyses: parent PTSD symptoms as a predictor of PR child symptom scores, controlling for CR PTSS (Model 1) and child PTSD diagnostic status (Model 2)*

|  | **Predictor** | ***B* (95% CI)^a^** | ***SE*** | ***β*** | ***p*** |
| --- | --- | --- | --- | --- | --- |
| *Model 1:* PR child PTSS, *F*(4, 114)=19.95, *p*<.001, *R^2^*=.41 | | | | | |
|  | Parent PDS Score | 0.56 (0.41, 0.71) | 0.07 | 0.60 | <.001 |
|  | CR PTSS | 0.14 (-0.01, 0.29) | 0.08 | 0.14 | .079 |
|  | Child Age | -0.02 (-0.16, 0.11) | 0.07 | -0.02 | .751 |
|  | Child Triage | 0.14 (-0.08, 0.37) | 0.12 | 0.09 | .245 |
| Model 2: PR child PTSS, *F*(4, 114)=25.23, *p*<.001, *R^2^*=.47 | | | | | |
|  | Parent PDS Score | 0.50 (0.35, 0.64) | 0.07 | 0.53 | <.001 |
|  | Diagnostic Status | 1.17 (0.50, 1.89) | 0.29 | 0.30 | <.001 |
|  | Child Age | -0.01 (-0.14, 0.11) | 0.06 | -0.01 | .887 |
|  | Child Triage | 0.13 (-0.08, 0.35) | 0.11 | 0.08 | .240 |

^a^ CI are bootstrapped with 10,000 replications

*Note.* CR: Child report. PDS: Posttraumatic Stress Diagnosis Scale. PR: Parent report. PTSS: Posttraumatic stress symptoms.

**Table 3.** *Regression analyses (n=70): prediction of child mean HR from CR and PR of child PTSS, separated by PTSD symptom cluster, controlling for child age*

|  | **Predictor** | ***B* (95% CI)^a^** | ***SE*** | ***β*** | ***p*** |
| --- | --- | --- | --- | --- | --- |
| *Model 1 (Mean HR, Cluster B):* *F*(3, 66)=2.68, *p*=.054, *R^2^*=.11 | | | | | |
|  | CR Intrusion | 0.13 (-0.05, 0.29) | 0.08 | 0.20 | .129 |
|  | PR Intrusion | 0.01 (-0.16, 0.21) | 0.09 | 0.01 | .952 |
|  | Child Age | -0.09 (-0.20, 0.02) | 0.06 | -0.21 | .105 |
| *Model 2 (Mean HR, Cluster C):* *F*(3, 66)=3.26, *p*=.027, *R^2^*=.13 | | | | | |
|  | CR Avoidance | 0.16 (-0.03, 0.39) | 0.09 | 0.22 | .084 |
|  | PR Avoidance | 0.07 (-0.13, 0.29) | 0.09 | 0.09 | .451 |
|  | Child Age | -0.08 (-0.19, 0.03) | 0.05 | -0.18 | .147 |
| *Model 3 (Mean HR, Cluster D):* *F*(3, 66)=4.51, *p*=.006, *R^2^*=.17 | | | | | |
|  | CR Hyperarousal | 0.25 (0.05, 0.44) | 0.10 | 0.30 | .012 |
|  | PR Hyperarousal | 0.05 (-0.12, 0.21) | 0.08 | 0.07 | .538 |
|  | Child Age | -0.09 (-0.19, 0.01) | 0.05 | -0.20 | .085 |

^a^ CI are bootstrapped with 10,000 replications

*Note*. CR: Child report. HR: Heart rate. PR: Parent report. SE: Standard error.

**Table 4.** *Regression analyses (n=70): prediction of child HFBP from CR and PR of child PTSS, separated by PTSD symptom cluster, controlling for child age*

|  | **Predictor** | ***B* (95% CI)^a^** | ***SE*** | ***β*** | ***p*** |
| --- | --- | --- | --- | --- | --- |
| *Model 1 (HFBP, Cluster B):* *F(3, 66)=1.79, p=.157, R^2^=.08* | | | | | |
|  | CR Intrusion | -0.17 (-0.31, -0.02) | 0.09 | -0.25 | .059 |
|  | PR Intrusion | -0.05 (-0.23, 0.10) | 0.10 | -0.07 | .585 |
|  | Child Age | -0.02 (-.14, .10) | 0.06 | -0.05 | .692 |
| *Model 2 (HFBP, Cluster C):* *F(3, 66)=2.01, p=.121, R^2^=.08* | | | | | |
|  | CR Avoidance | -0.12 (-0.32, 0.09) | 0.10 | -0.15 | .248 |
|  | PR Avoidance | -0.18 (-0.37, -0.01) | 0.10 | -0.23 | .068 |
|  | Child Age | -0.02 (-0.13, 0.11) | 0.06 | -0.04 | .772 |
| *Model 3 (HFBP, Cluster D):* *F(3, 66)=4.24, p=.008, R^2^=.16* | | | | | |
|  | CR Hyperarousal | -0.14 (-0.51, -0.14) | 0.10 | -0.36 | .003 |
|  | PR Hyperarousal | -0.10 (-0.27, 0.07) | 0.09 | -0.14 | .252 |
|  | Child Age | -0.02 (-0.12, 0.09) | 0.05 | -0.04 | .726 |

^a^ CI are bootstrapped with 10,000 replications

*Note*. CR: Child report. HFBP: High-frequency band power. PR: Parent report. SE: Standard error.

**Table 5.** *Regression analyses (n=70): prediction of child LFBP from CR and PR of child PTSS, separated by PTSD symptom cluster, controlling for child age*

|  | **Predictor** | ***B* (95% CI)^a^** | ***SE*** | ***β*** | ***p*** |
| --- | --- | --- | --- | --- | --- |
| *Model 1 (LFBP, Cluster B):* *F(3, 66)=4.19, p=.009, R^2^=.16* | | | | | |
|  | CR Intrusion | -0.23 (-0.39, -0.08) | 0.08 | -0.35 | .006 |
|  | PR Intrusion | -0.02 (-0.21, 0.16) | 0.09 | -0.03 | .841 |
|  | Child Age | 0.04 (-0.07, 0.15) | 0.06 | 0.09 | .471 |
| *Model 2 (LFBP, Cluster C):* *F(3, 66)=2.08, p=.112, R^2^=.09* | | | | | |
|  | CR Avoidance | -0.17 (-0.35, -0.01) | 0.10 | -0.22 | .095 |
|  | PR Avoidance | -0.03 (-0.22, 0.16) | 0.10 | -0.04 | .766 |
|  | Child Age | 0.06 (-0.06, 0.16) | 0.06 | 0.12 | .333 |
| *Model 3 (LFBP, Cluster D):* *F(3, 66)=3.77, p=.015, R^2^=.15* | | | | | |
|  | CR Hyperarousal | -0.24 (-0.43, -0.07) | 0.10 | -0.28 | .022 |
|  | PR Hyperarousal | -0.10 (-0.26, 0.01) | 0.09 | -0.14 | .241 |
|  | Child Age | 0.06 (-0.04, 0.16) | 0.05 | 0.13 | .288 |

^a^ CI are bootstrapped with 10,000 replications

*Note*. CR: Child report. LFBP: Low-frequency band power. PR: Parent report. SE: Standard error.
